# Inharmonic speech reveals the role of harmonicity in the cocktail party problem

**DOI:** 10.1038/s41467-018-04551-8

**Published:** 2018-05-29

**Authors:** Sara Popham, Dana Boebinger, Dan P. W. Ellis, Hideki Kawahara, Josh H. McDermott

**Affiliations:** 1 Department of Brain and Cognitive Sciences, MIT, Cambridge, MA 02139 USA; 2 Helen Wills Neuroscience Institute, UC Berkeley, Berkeley, CA 94720 USA; 3 Program in Speech and Hearing Sciences, Harvard University, Cambridge, MA 02138 USA; 4 Google Research, New York, NY 10011 USA; 5 Wakayama University, Wakayama, Sakaedani 930 Japan

## Abstract

The “cocktail party problem” requires us to discern individual sound sources from mixtures of sources. The brain must use knowledge of natural sound regularities for this purpose. One much-discussed regularity is the tendency for frequencies to be harmonically related (integer multiples of a fundamental frequency). To test the role of harmonicity in real-world sound segregation, we developed speech analysis/synthesis tools to perturb the carrier frequencies of speech, disrupting harmonic frequency relations while maintaining the spectrotemporal envelope that determines phonemic content. We find that violations of harmonicity cause individual frequencies of speech to segregate from each other, impair the intelligibility of concurrent utterances despite leaving intelligibility of single utterances intact, and cause listeners to lose track of target talkers. However, additional segregation deficits result from replacing harmonic frequencies with noise (simulating whispering), suggesting additional grouping cues enabled by voiced speech excitation. Our results demonstrate acoustic grouping cues in real-world sound segregation.

## Abstract (web summary)

Harmonicity is associated with a single sound source and may be a useful cue with which to segregate the speech of multiple talkers. Here the authors introduce a method for perturbing the constituent frequencies of speech and show that violating harmonicity degrades intelligibility of speech mixtures.

## Introduction

Auditory scenes with multiple sound sources are ubiquitous in our lives, and the ability to segregate a particular source of interest from the sound mixture that enters the ears is critical for communication and recognition^1–8^. Humans with normal hearing can usually segregate sounds successfully, helping us solve the “cocktail party problem.” Although spatial information contributes to our success in this domain^9–13^, humans segregate sounds remarkably well from monaural signals, as when listening to mono music. This is possible because natural sounds exhibit statistical regularities that the brain can use to group acoustic energy that is likely to have originated from the same source. Understanding sound segregation thus requires uncovering the statistical regularities of natural sounds and the processes that make use of them.

Prior studies of sound segregation, driven by intuitions about the structure of natural sounds, have indicated the importance of a small number of acoustic grouping cues: common onset^14, 15^, harmonicity^16–20^, and repetition^21, 22^. Although these sound properties presumably derive their importance from natural sound statistics, they have been studied primarily using relatively simple artificial sounds. Because speech, music, and other everyday sounds are more complex and varied than the artificial stimuli used in most psychoacoustic studies of acoustic grouping, it is not obvious whether effects observed with synthetic stimuli will transfer to real-world conditions.

The goal of the present study was to explore the role of harmonicity in the segregation of natural speech. Harmonicity refers to the situation in which sound frequencies are integer multiples of a common fundamental frequency (f0). Harmonicity is believed to underlie pitch perception^23^ and musical harmony^24^ and may be detected by single neurons in the primate auditory system^25^. Because harmonic frequencies typically result from a single sound-generating process that is periodic in time, their presence also provides a cue that they were generated by a common source. We sought to test whether the classic psychoacoustic grouping effects of harmonicity on synthetic tones^16–19^ would replicate with speech and whether harmonicity would be critical for extracting speech information from mixtures of talkers.

The main obstacle to investigating sound segregation with natural sounds such as speech has been the difficulty of manipulating such sounds for experimental purposes. Prior attempts to explore speech segregation have relied on relatively limited synthetic approximations. Numerous studies have examined the perception of concurrent synthetic vowels and support a role for harmonicity in their segregation^26–29^. But to our knowledge, there has been only one attempt to synthesize inharmonic speech utterances (words, phrases, sentences, etc.) for segregation experiments, and the limitations of resynthesis necessitated the use of speech with a flattened pitch contour^30^.

**Fig. 1:**
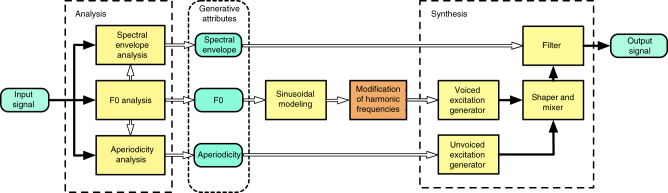
Schematic of STRAIGHT with sinusoidal modeling. An input signal is decomposed into a time-varying spectral envelope and time-varying periodic and aperiodic excitation. The periodic excitation is modeled as a sum of sinusoids, the frequencies of which can be altered and combined with the aperiodic (unvoiced) excitation and then filtered to impose the original spectrotemporal envelope, yielding inharmonic speech

To address these challenges, we utilized an extension of the STRAIGHT methodology for speech analysis and synthesis^31^. STRAIGHT is an algorithm for separately estimating the time-varying source and filter that underlie a speech signal. We modified the conventional STRAIGHT algorithm to enable the synthesis of speech signals with inharmonic carrier spectra that are otherwise natural^32^. We modeled the time-varying source (the “excitation”) as a sum of sinusoidal components that could then be individually perturbed and recombined with the original time-varying spectrotemporal filter, yielding inharmonic speech (Fig. 1). Demonstrations of the resulting stimuli can be found online at: http://mcdermottlab.mit.edu/inharmonic_speech_examples/.

We used this new speech synthesis tool to test the role of harmonicity in the grouping and segregation of natural speech. We first mistuned individual harmonics of a speech utterance, finding that this caused the mistuned harmonic to be heard as a separate “whistle” concurrent with the rest of the utterance—an analog of the classic harmonic mistuning effect with complex tones^16, 17^. We then altered the harmonicity of speech utterances in cocktail party scenarios by replacing harmonic excitation with either inharmonic frequency components or noise (simulating whispering). We found that inharmonic speech was less intelligible in mixtures or when superimposed on speech babble, even though intelligibility was comparable for single utterances in quiet. Moreover, when listeners were instructed to attend to a particular talker, inharmonic excitation caused listeners to erroneously report words from the competing talker, as though they lost track of the target talker. These results suggest that harmonicity contributes to the grouping and streaming of natural speech, validating the real-world relevance of prior findings with artificial synthetic stimuli. However, segregation deficits were substantially worse when harmonic excitation was replaced with noise, suggesting additional benefits of the discrete frequencies that result from harmonic excitation. The results demonstrate a methodology for studying acoustic grouping in real-world sounds and help to reveal the richness of real-world sound segregation.

## Results

### Harmonic mistuning

**Fig. 2:**
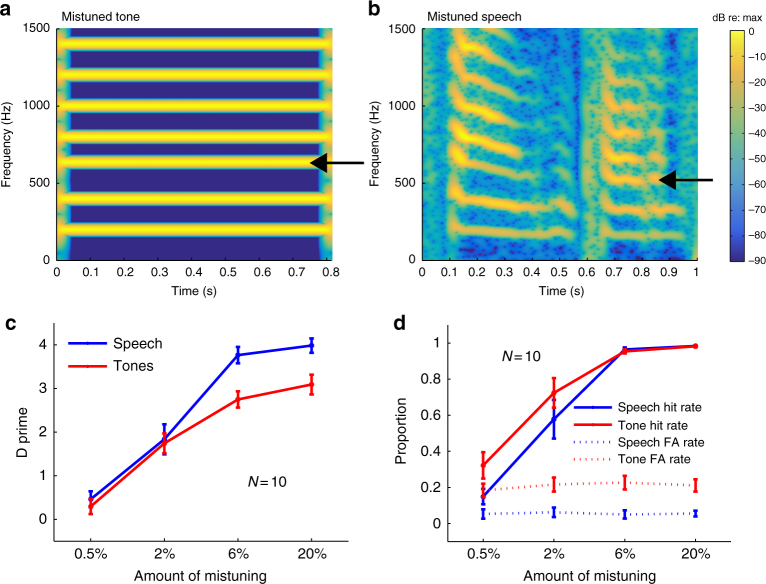
Detection of mistuning of the third harmonic in speech utterances and in complex tones (Experiment 1). **a** Spectrogram of example tone stimulus with the third harmonic mistuned upward by 6%. Arrow indicates mistuned harmonic. **b** Spectrogram of example speech stimulus with the third harmonic mistuned upward by 6%. Arrow indicates mistuned harmonic. **c** Sensitivity to mistuning in tones and speech. **d** Hit and false alarm rates for tones and speech. Hits were defined as correct responses on trials in which the harmonic was mistuned, and false alarms were defined as incorrect responses on trials in which the harmonic was not mistuned. False alarm rates were calculated from the half of the trials in which there was no mistuning, randomly assigned to the four conditions. Error bars here and in **c** plot SEM

We began by testing whether the classic harmonic mistuning effect would occur for speech. For synthetic tones, mistuning a harmonic by a few percent (Fig. 2a) is sufficient to cause it to be heard as a separate sound^16, 17^. A priori, it was not obvious what to expect from analogous manipulations with speech. It seemed plausible that the additional structure present in speech relative to synthetic tones might attenuate the effects of mistuning, because there are other cues indicating that the harmonics belong to the same source. Alternatively, one might expect a particularly strong prior presumption that the frequencies composing speech are harmonic, potentially producing a stronger effect of mistuning.

We resynthesized speech with either the harmonic frequencies unperturbed or with the third harmonic mistuned by varying amounts (Fig. 2b) and asked listeners whether they heard one or two sounds. For comparison, listeners performed an analogous task with synthetic tones (in separate blocks, counterbalanced for order). In both cases, the task replicated the experimental paradigm used in the original study by Moore and colleagues^16^. The third harmonic was selected for mistuning because this produced consistent mistuning detection thresholds in these original experiments. In the tone conditions, mistuning produced the subjective impression of two tones—a complex tone coupled with a fainter pure tone. In the speech conditions, when the mistuned harmonic segregated from the rest of the speech utterance, it sounded like a faint whistle concurrent with the speech (because whistling typically produces a frequency- and amplitude-modulated pure tone, similar to an isolated harmonic of a spoken utterance).

As shown in Fig. 2c, once the perturbed harmonic was sufficiently mistuned, listeners reliably detected it in both speech and tones (producing a significant main effect of the amount of mistuning, *F*(3,27) = 65.17, *p* < 0.0001). Thresholds (e.g., the mistuning amount producing a *d*’ of 2) were in the vicinity of 2% in both cases, but sensitivity was better for speech once the mistuning was above threshold (producing a main effect of stimulus type: *F*(1,27) = 13.18, *p* = 0.0055, and an interaction with mistuning amount: *F*(3,27) = 6.77, *p* = 0.0015). Inspection of the hit and false alarm rates (Fig. 2d) indicates that this results from listeners being more likely to report hearing two tones when all frequencies of a complex tone were in fact harmonic (*t*(9) = 4.20, *p* = 0.002; hit rates also appear a bit higher for tones, but the comparison is impaired by a ceiling effect for the larger mistuning amounts). Consistent with this finding, we noted subjectively that we sometimes had the sense of hearing more than one tone in the complex tone conditions even when there was no mistuning, perhaps due to audibility of individual harmonics^33^. The analogous phenomenon never occurred for the speech stimuli. This could reflect a stronger prior for harmonic structure in speech than for synthetic tones, perhaps because we have a lifetime of exposure to harmonic speech.

### Concurrent words

The harmonic mistuning effect for speech found in Experiment 1 suggests that harmonicity contributes to the grouping of individual speech utterances. However, the most important consequence of acoustic grouping is arguably the extraction of speech information from mixtures of utterances, as in the canonical cocktail party problem. To explore whether any acoustic grouping effects of harmonicity would aid cocktail party listening, we synthesized speech with either inharmonic or harmonic carrier spectra, presented listeners with mixtures of two utterances, and asked them to report what was said. If harmonicity is critical to correctly estimating the content of a speech utterance from a mixture, one would expect mixture intelligibility to be impaired for inharmonic speech.

We first measured intelligibility for single words and mixtures of concurrent words. The words were excerpted from sentences from the TIMIT speech corpus. Excerpted words were used instead of single isolated words because we wanted to subsequently conduct experiments with sentences of the same material. Participants typed as many words as they could understand (up to two words on mixture trials). Responses were scored by the experimenter, blind to the condition. We sought to answer three questions: (1) whether inharmonic speech would be more difficult to understand than normal (harmonic) speech when presented in mixtures, (2) whether we could maximize any such performance decrement by parametrically increasing the degree of inharmonicity, and (3) whether any effect on speech mixtures could be explained by an effect on intelligibility of utterances in quiet. In the mixture conditions, we always mixed utterances of the same type (i.e., harmonic with harmonic or inharmonic with inharmonic) in order to compare natural listening conditions, in which all sources are harmonic, to hypothetical conditions in which all sources would be inharmonic. The two words in each mixture were produced by different speakers of the same gender, as this created a challenging listening situation that seemed likely to create a strong test of the role of harmonicity.

**Fig. 3:**
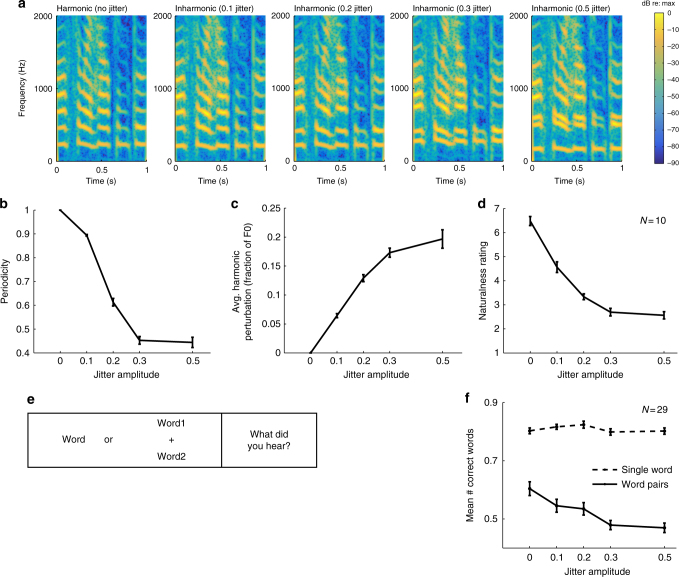
Effect of inharmonicity on concurrent word segregation (Experiment 2). **a** Spectrogram of excerpts of harmonic and inharmonic speech with harmonics jittered by 0%, 10%, 20%, 30%, and 50% of the f0, respectively. **b** Waveform aperiodicity (the height of the peak of the autocorrelation function) for each jitter condition. **c** Average absolute harmonic perturbation for each jitter condition. Error bars here and in **b** plot standard deviation. **d** Ratings of naturalness of spoken sentences for different degrees of inharmonicity (*N* = 10). **e** Schematic of trial structure for word segregation task. **f** Intelligibility of single words and concurrent word pairs as a function of the degree of inharmonicity (*N* = 29). Error bars here and in **d** plot SEM

We generated inharmonic carrier spectra by jittering each harmonic by a random proportion of the f0^34^. We parametrically varied the degree of inharmonicity by constraining these proportions to lie within a range that varied from 0 (producing perfectly harmonic speech) to +/−50% of the f0 (Fig. 3a). To minimize the chances that inharmonicity would introduce beating that might influence intelligibility for reasons unrelated to grouping (by altering the spectrotemporal envelope that conveys speech content), we constrained the jitter patterns to not produce pairs of harmonics that were closer than 30 Hz to each other. As a result, beat frequencies were never less than 30 Hz, modulation frequencies above which contribute little to speech intelligibility^35^. To help ensure that the resulting jitter patterns were as inharmonic as possible subject to these constraints (i.e., to avoid random jitters that by chance would produce approximately harmonic patterns), we generated 10,000 jitter patterns for each condition. We then used the patterns that were maximally inharmonic. Because there is no standard measure of the degree of inharmonicity, we instead measured its time-domain analog (aperiodicity), selecting the frequency patterns that minimized the autocorrelation peak height (for complex tones synthesized with the jitter pattern). Figure 3b, c show that increasing the jitter amplitude subject to these constraints indeed increases aperiodicity and the average harmonic perturbation, at least up to a point.

To assess the subjective effect of increasing inharmonicity, we asked participants to rate the naturalness of speech exemplars. As shown in Fig. 3d, ratings of speech naturalness decreased with the degree of inharmonicity (main effect of jitter amplitude: *F*(4,36) = 158.37, *p* < 0.0001). Naturalness appears to level off at 30% jitter (the difference between naturalness of 30 and 50% jitter was not significant: *t*(9) = 1.24, *p* = 0.25).

The intelligibility of concurrent words (Fig. 3e, f) similarly decreased with the jitter amplitude (*F*(4,112) = 18.07, *p* < 0.0001) up to 30% (*t*(28) = 0.52, *p* = 0.61, comparison of performance between 30 and 50% jitter). These results are consistent with the effects of jittering on the physical correlates of aperiodicity (Fig. 3b, c), which did not increase much past 30%. The results are also consistent with a prior study that found that the pitch shift induced by a mistuned harmonic of a complex tone dissipated as the other harmonics were jittered and was nearly eliminated by 30% jitter^34^.

By contrast, inharmonicity did not significantly affect the intelligibility of single words in quiet (Fig. 3f; *F*(4,112) = 2.17, *p* = 0.08), producing an interaction between task and inharmonicity (*F*(4,28) = 12.98, *p* < 0.0001). The sub-ceiling performance for single words in quiet is likely due to the variability of the TIMIT corpus along with the words having been excerpted from sentences, which, due to effects of coarticulation, somewhat reduces intelligibility. Overall, these data are consistent with the subjective impression of listening to examples of inharmonic speech: inharmonic utterances are obviously unnatural but nonetheless seem fully intelligible, though they are somewhat less fused than harmonic utterances.

To ensure that the limited effect of inharmonicity was not an artifact of constraining harmonics to stay at least 30 Hz apart, we conducted control experiments in which this constraint was removed (Supplementary Fig. 1). We simply selected the jitter patterns that were most aperiodic for each perturbation magnitude without regard for the proximity of harmonics, regenerated stimuli, and repeated the experiments of Fig. 3d, f. Unlike the results with the 30 Hz constraint (Fig. 3f), the intelligibility of single words in quiet was affected by frequency jitter for these unconstrained stimuli (Supplementary Fig. 1; *F*(4, 56) = 4.7415, *p* = 0.0023). This result is consistent with the idea that beating between adjacent frequency components can impair intelligibility for reasons unrelated to grouping and provides support for the constrained manipulation that we employed in the main experiments. However, naturalness ratings and mixture intelligibility again appeared to level off after 30% even without the 30 Hz constraint. Collectively, the results suggest that our parametric jitter manipulation succeeded in maximizing the effect of inharmonicity on grouping. Despite this, we observed only a modest (though highly significant) effect; intelligibility in mixtures clearly does not completely fall apart.

### Speech in noise

**Fig. 4:**
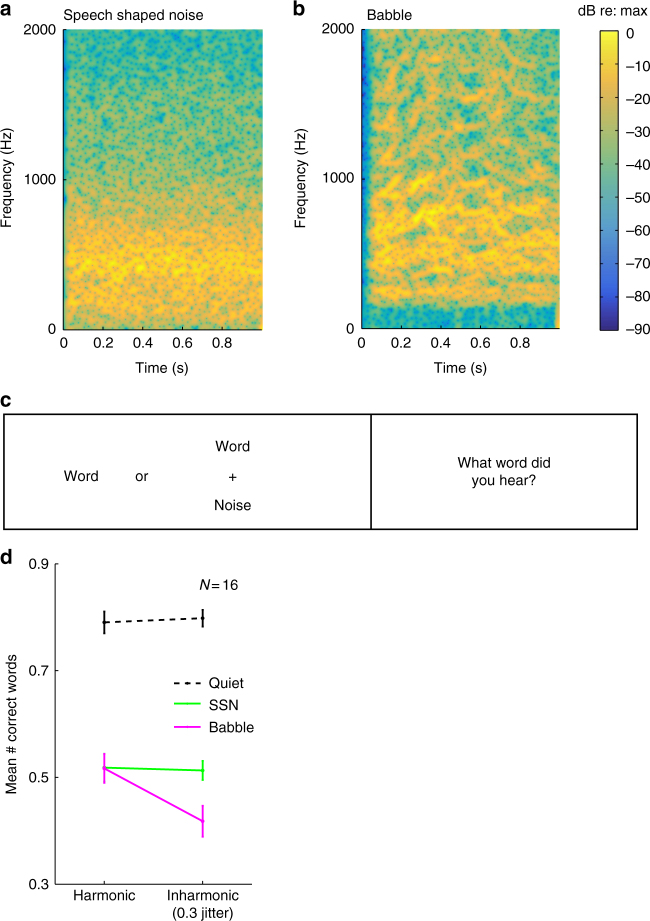
Intelligibility of harmonic and inharmonic speech in speech-shaped noise and babble (Experiment 3). **a** Spectrogram of example of speech-shaped noise. **b** Spectrogram of example of babble (20 talkers). **c** Schematic of trial structure for word recognition task. **d** Word recognition in quiet, speech-shaped noise, and babble. Error bars denote SEM

The results with concurrent words in Experiment 2 are consistent with an effect of inharmonicity that is mediated by its effect on grouping, in that inharmonicity produced a substantial effect on intelligibility for mixtures but not for single words. However, performance was fairly high overall for single words, and it seemed important to investigate whether inharmonicity would produce an effect if intelligibility was lowered by presenting words in noise. We used both speech-shaped noise (SSN; Fig. 4a) and babble (Fig. 4b), with signal-to-noise ratios (SNRs) that were adjusted to substantially reduce overall performance in both cases (−4 dB for SSN and +4 dB for babble), and measured intelligibility for harmonic and inharmonic speech in quiet as well as in both types of noise (Fig. 4c). The SSN was white noise filtered to have the talker’s power spectrum. We generated babble by summing utterances from 20 different talkers of both genders. The logic was that SSN does not introduce much ambiguity about how voiced speech should be grouped, because sound energy from noise is generally not confused with sound energy from speech^36^. In contrast, babble contains audible glimpses of individual voices; these voice fragments must be segregated from the target utterance, and it seemed plausible that there would be a significant grouping ambiguity (Fig. 4b). If the effect of inharmonicity is related to a general reduction of intelligibility, one would expect performance to be impaired for both types of noise, but if inharmonicity instead primarily affects intelligibility via its effect on how sound is grouped, the effect might be limited to babble.

As is evident in Fig. 4d, presenting words in SSN reduced the intelligibility of both harmonic and inharmonic speech by approximately equal amounts—performance remained indistinguishable for the two conditions (*t*(15) = 0.22, *p* = 0.83). In contrast, intelligibility in babble was reduced more for inharmonic than for harmonic speech (*t*(15) = 3.36, *p* = 0.004), producing an interaction between the type of speech and the type of background noise (*F*(1,15) = 5.68, *p* = 0.031). These results provide further evidence that inharmonicity specifically affects the ability of listeners to group the frequencies of speech, in that it apparently does not affect the intelligibility of speech so long as grouping is relatively unambiguous, even when listening conditions are adverse.

### Noise excitation

**Fig. 5:**
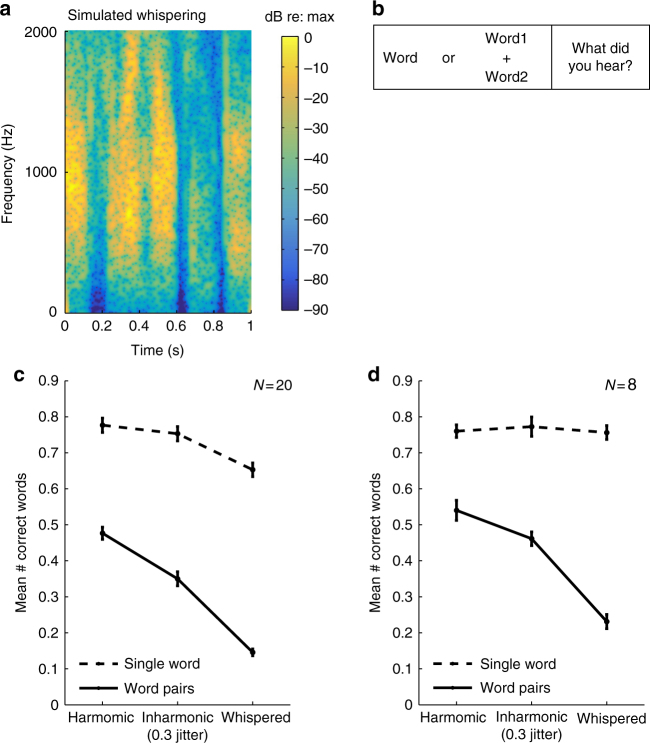
Effect of noise excitation, simulating whispering (Experiment 4). **a** Spectrogram of utterance from Fig. 3a resynthesized with noise excitation. **b** Schematic of task. **c** Intelligibility of mixtures of harmonic, inharmonic, and noise-excited words, along with that of single words in quiet for each condition. Here and in **d**, error bars denote SEM. **d** Intelligibility of mixtures of harmonic, inharmonic, and noise-excited words after equating intelligibility of single words in quiet

To better understand the relatively modest effect of making speech inharmonic, we conducted an experiment in which we replaced harmonic excitation with synthetic breath noise, simulating whispering (Fig. 5a). Unlike real-world whispering^37, 38^, these synthetic stimuli were generated with the same spectrotemporal envelope used to synthesize harmonic and inharmonic speech. By comparing intelligibility of harmonic, inharmonic, and whispered speech mixtures (Fig. 5b), we hoped to test whether harmonic speech excitation might have benefits other than harmonicity per se, perhaps related to the presence of discrete frequency components, which are not present for noise excitation.

As shown in Fig. 5c, noise excitation impaired intelligibility of concurrent words far more than did inharmonicity, but also reduced intelligibility of single words in quiet compared to harmonic or inharmonic speech (*t*(19) = 5.10, *p* < 0.0001, paired *t*-test between harmonic and noise-excited words in quiet). This latter finding is consistent with prior findings with natural whispering^39^ and complicates the interpretation of the deficits for concurrent words, which could arguably reflect heightened effects of intrinsic intelligibility differences. One prior study found intelligibility deficits for concurrent whispered vowels^40^ but also left open the contribution of intelligibility differences in quiet.

In order to more conclusively test the effect of simulated whispering on the perception of speech mixtures, we sought to equate performance for single words in quiet. We accomplished this by first measuring the intelligibility of a large set of words in isolation. We then sorted the words according to how well they were recognized and assigned the easiest words to the whispered condition, dividing a remaining subset randomly between the harmonic and inharmonic conditions (see Methods for details).

As shown in Fig. 5d, we succeeded in equating the intelligibility of all three types of speech in quiet (*F*(2,14) = 0.204, *p* = 0.82). However, large differences between conditions were nonetheless evident for mixtures of utterances. Inharmonicity again had a significant but modest effect on intelligibility in mixtures (*t*(7) = 3.2, *p* = 0.01), but the effect of simulated whispering (*t*(7) = 10.0, *p* < 0.0001; paired *t*-test between inharmonic and whispered intelligibility) was much larger (*t*-test on differences between conditions: *t*(7) = 3.43, *p* = 0.011; harmonic–inharmonic vs. inharmonic–whispered). Listeners correctly perceived one of the two words in a mixture on only ~1 out of every 5 trials. This result suggests that there are segregation benefits of voiced speech excitation other than harmonicity per se, in that maximally inharmonic speech remains far easier to extract from speech mixtures than does noise-excited speech.

### Concurrent sentences

**Fig. 6:**
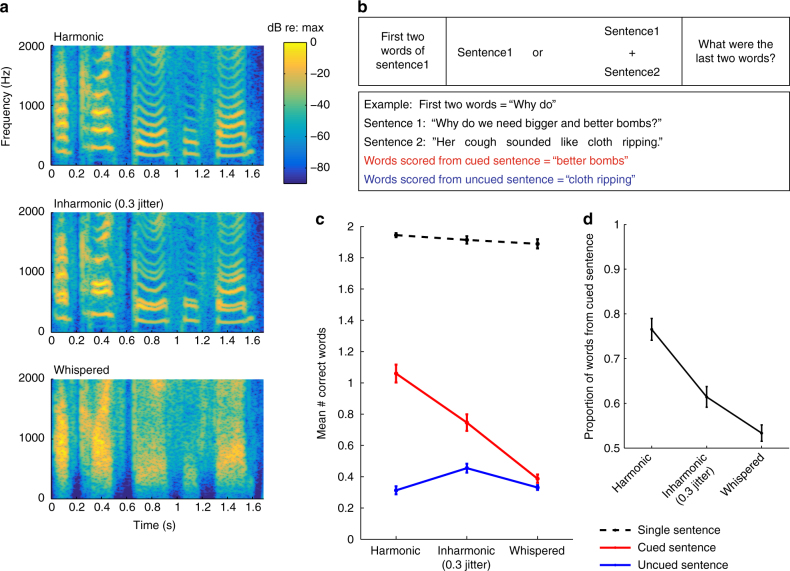
Effect of inharmonicity and noise excitation on the segregation and streaming of concurrent sentences (Experiment 5). **a** Spectrograms of an example sentence synthesized with harmonic, inharmonic, and noise excitation. **b** Schematic of sentence intelligibility tasks. Listeners were shown the first two words of the cued sentence and asked to type in the last two words. **c** Effect of inharmonicity and simulated whispering on concurrent sentence intelligibility. Error bars here and in **c** denote SEM. **d** The proportion of words correctly reported per trial that were from the uncued sentence

To test whether comparable effects of inharmonicity and noise excitation would be evident in more realistic conditions, we presented listeners with concurrent sentences (Fig. 6a). At the start of each trial, listeners were visually presented with the first two words of one of the sentences; they were instructed to attend to that sentence and report its last two words (Fig. 6b). We separately scored words correctly reported from the cued sentence as well as words mistakenly reported from the uncued sentence (because these might indicate that listeners had lost track of the cued voice). Single sentences were also presented for comparison.

As shown in Fig. 6c, when only words from the cued sentence were counted (red curve), there was a performance decrement for inharmonic compared to harmonic speech (*t*(14) = 6.98, *p* < 0.0001) and another decrement for simulated whispered speech compared to inharmonic (*t*(14) = 12.82, *p* < 0.0001), producing a main effect of speech type (*F*(2,28) = 7.28, *p* < 0.0001). This result is a qualitative replication of the results of Experiments 2 and 4 (with sentences instead of single words). A different pattern of results was evident for words reported from the uncued sentence. Listeners were more likely to report words from the uncued voice when the speech was inharmonic than when it was harmonic (*t*(14) = 4.92, *p* < 0.001), producing an interaction between the effect of speech type and the sentence being scored (*F*(2,28) = 49.55, *p* < 0.0001). This finding is consistent with the idea that harmonicity helps listeners to track speech utterances over time, potentially via pitch. When harmonicity is disrupted, listeners appear more likely to mistakenly switch their attentional focus onto a competing talker, which remains somewhat identifiable despite the inharmonicity.

Whispering also greatly impaired the ability to track the target talker—listeners were not significantly more likely to report words from the cued than the uncued sentence (*t*(14) = 2.1, *p* = 0.055). These results are summarized in Fig. 6d, which plots the proportion of correctly reported words that came from the cued sentence. When speech was harmonic, most correctly reported words were from the cued talker, indicating successful selection of the target speech stream, but this proportion was lower for inharmonic and noise-excited speech (*F*(2,28) = 37.87, *p* < 0.0001).

## Discussion

We rendered speech inharmonic in order to investigate the role of harmonicity in the grouping and streaming of natural speech. We observed four effects that appear to reflect grouping and/or streaming. First, mistuning one of the harmonics of a speech signal caused the harmonic to be heard as a distinct sound source (resembling whistling), qualitatively replicating the classic findings with mistuned harmonics of complex tones. Second, mixtures of inharmonic words were less intelligible than mixtures of harmonic words (by about 20%). Third, inharmonicity had no measurable effect on the intelligibility of words in quiet, or in stationary noise, but reduced intelligibility for words presented in babble. These latter two sets of findings suggest that the effect of inharmonicity is due to its effect on grouping rather than on some more general degradation of speech information. Fourth, when attending to one of two concurrent sentences, listeners were more likely to report the words of the talker they were instructed to ignore when speech was inharmonic, suggesting that harmonicity helps to track target talkers over time. Although all four of these effects were reliable and statistically significant, larger effects occurred when we simulated whispering by replacing tonal excitation with noise. Noise-excited words were typically unintelligible in mixtures even when their intelligibility in quiet was equated to that of harmonic words. Overall, the results support a role for harmonicity in grouping and streaming speech but suggest consequences of voiced speech excitation other than harmonicity per se, perhaps related to the presence of discrete frequencies inherent to periodic excitation.

Our methodology leveraged the high-fidelity estimates of source and filter provided by STRAIGHT, which permits speech excitation to be altered while preserving the spectrotemporal envelope that conveys speech information. By modeling the excitation as a sum of sinusoidal harmonics, we were able to perturb the frequencies of individual harmonics, specifically disrupting harmonic frequency relations without creating other differences between the harmonic and inharmonic stimuli. A natural concern with any speech manipulation is whether the effects of the manipulation could somehow reflect artifacts of the synthesis procedure. One reason to think that this is not the case here is that the inharmonicity manipulation appears to not reduce intelligibility in the absence of multiple talkers. Artifacts that might reduce intelligibility more generally would be expected to affect performance in quiet or in stationary noise, but we observed little effect in either case. The relatively clean manipulation of harmonicity was made possible by the STRAIGHT signal processing framework, which provides an estimate of the spectrotemporal envelope with minimal interference from the underlying carrier signal^31^. Our subjective impression is that subtle artifacts are often audible in the resynthesized speech utterances but that these are similar for harmonic and inharmonic excitation, and thus do not account for the effects of inharmonicity described here.

The methodological innovation enabling the manipulation of natural speech was critical to our key scientific results. The finding that inharmonicity produces a modest effect is only meaningful in the context of an ecologically valid open set recognition task, which can only be performed with natural speech. Moreover, the larger deficits seen with whispering can only be attributed to segregation problems if baseline intelligibility in quiet is matched, which depended on the use of large speech corpora (in order to choose sets of stimuli for which intelligibility was matched across conditions), which in turn required being able to manipulate natural speech.

To our knowledge, our results provide the first measurements of the effects of harmonicity on the cocktail party problem with natural speech. However, many of our experiments represent extensions of classic results with synthetic stimuli. Our results are consistent with the classic literature but demonstrate several additional effects that could only be obtained using natural speech stimuli.

The results of mistuning a single harmonic of a speech utterance were qualitatively similar to those of the analogous experiment with complex tones^16, 17^. For both speech and tones, thresholds for detecting a mistuned harmonic were approximately 1–2%. However, sensitivity in the two cases was not identical: listeners were better at detecting mistuning in speech once the mistuning was above threshold, largely because they sometimes mistakenly hear harmonics within complex tones as distinct even though they are not mistuned. It thus seems plausible that internal models of speech could be accentuating the effect of the mistuning due to strong priors on the nature of speech excitation.

Our speech intelligibility results are qualitatively consistent with earlier experiments with double vowels^27, 29^ and with pitch-flattened speech^30^, which reported recognition impairments for inharmonic carriers. Our experiments demonstrate the effect in natural speech with time-varying pitch but show that it is modest in size even when measured in relatively realistic open set recognition tasks.

The finding that listeners are more likely to lose track of a target talker when their speech is made inharmonic is consistent with prior results demonstrating the importance of pitch differences to the ability to attend to a target talker^41–43^. Our results suggest that the effect is mediated by f0-based pitch rather than, say, the overlap between the talker spectra, which should be similar for harmonic and inharmonic speech.

Our results with simulated whispering clarify results from previous work on noise-vocoded speech. In general, the intelligibility of noise-vocoded speech in the presence of concurrent sources is poor^44–47^, consistent with our results. Our results suggest that segregation impairments with noise-vocoded speech are not primarily due to the distortion induced to the spectrotemporal envelope by the small number of frequency channels used, because our noise-excited speech stimuli were generated from the same spectrotemporal envelope as the harmonic/inharmonic speech.

Our results also clarify prior work with whispered speech, suggesting that intelligibility deficits for concurrent whispered vowels^40^ or of whispered sentences in different types of masking noise^48^ cannot be explained by impaired intelligibility in quiet. To our knowledge, this issue has not been widely considered in the prior literature but is an effect that theories of sound segregation must consider and account for. Moreover, the segregation deficits for noise-excited speech could help to explain the nature of mammalian vocalizations (as voiced rather than noise-excited).

Relative to the prior literature with simple synthetic stimuli, our work thus makes two main contributions. First, the experiments confirm several conclusions from classic work that employed simpler stimuli. The community has engaged in such work with the faith that such effects have real-world relevance, and our experiments provide some validation of this approach. The results also give plausibility to the idea that grouping from harmonicity could derive from the need to segregate speech. This hypothesis is consistent with the presence of harmonic mistuning effects in native Amazonians^49^ who have little experience with polyphonic music (arguably the other everyday situation in which harmonicity is likely to be most critical for sound segregation). Second, experiments with real-world stimuli lead to new conclusions that would not have been possible without stimuli based on natural speech; namely, that effects of inharmonicity on speech segregation are modest and that effects of noise excitation on speech segregation are large. A future contribution of the work may also lie in the fact that the ultimate goal of theories of sound segregation is to explain human performance with real-world sounds. The effects described here represent strong tests for next-generation models in this domain.

Although the effects of inharmonicity on mixture intelligibility were statistically significant, they were modest. Prior to conducting the experiments, we had imagined that inharmonic speech might fall apart into a sea of individual harmonics when presented concurrently with another speech signal, rendering it unintelligible, but that is manifestly not the case. The modest nature of the effects was not due to the constraints we imposed on inharmonicity, because unconstrained jitter patterns produced similarly modest effects (Supplementary Fig. 1). It is also noteworthy that the harmonic perturbations in our jitter conditions exceeded the amount necessary to cause a single mistuned harmonic to segregate from a speech signal (compare Figs. 2a and 3b), and it is thus no surprise that inharmonic speech sounds less fused than harmonic speech. The situation is somewhat analogous to that of sine-wave speech^50^, in which sinusoidal components can support the perception of speech despite not sounding like a single source. Unlike sine-wave speech^51^, inharmonic speech remains largely intelligible in mixtures, perhaps because other grouping cues remain present or because the overall evidence for speech structure is stronger.

What explains the modest effect of inharmonicity? One possibility is that other grouping cues, such as the co-modulation of frequency components^15, 52^, serve to group frequencies even when they are not harmonic. This hypothesis is consistent with theories that posit an important role for the coherence of temporal fluctuations across frequency channels^53, 54^. The importance of co-modulation could potentially also explain the deleterious effects of noise excitation, which presumably increases the overlap of concurrent speech signals within frequency channels, reducing or eliminating the co-modulation they would otherwise produce. Another related possibility is that the spectral sparsity of harmonic or inharmonic excitation allows matching of a speech mixture to stored speech models^55–57^, which themselves enable grouping. The possibility of “glimpsing”^58^ in the spectral dips between resolved harmonics has also been suggested by experiments in which speech was presented concurrently with harmonic or inharmonic tones^59^.

The ability to manipulate individual frequency components of speech excitation while preserving the spectrotemporal envelope should facilitate the investigation of many other related questions. One open question is whether common frequency modulation (FM) induces grouping. Psychoacoustic studies with synthetic stimuli have generally failed to find clear evidence for effects of common FM on grouping^60, 61^, but it is possible that any effects would be specific to more realistic stimuli. Our methodology could be straightforwardly extended to investigate this issue.

It should also be possible to use inharmonic speech to investigate the mechanisms underlying grouping by harmonicity in real-world conditions. Previous experiments have found that when one of two concurrent sounds is harmonic and the other inharmonic, performance is better when the target sound is inharmonic and the background harmonic^28, 62^, potentially indicative of “harmonic cancellation” mechanisms. We did not investigate such asymmetries here, but our methods could permit similar questions to be asked of natural speech. It could also be diagnostic to test alternative forms of inharmonicity^63^ with natural speech. More generally, inharmonic speech holds promise as a tool for studying real-world pitch perception in speech and singing^64^.

The impairments induced by whispering in sound mixtures merit further study. As discussed above, the effects could implicate the spectral sparsity provided by discrete frequency components, which are absent in whispering. Alternatively, excitation with discrete frequency components could itself provide a grouping cue via the characteristic alternation that normally occurs between voiced and unvoiced excitation. This alternation would be largely eliminated by whispering. Extensions of our synthesis methodology could be used to test these possibilities.

Although the underlying causes remain to be determined, it seems noteworthy that the effects of noise excitation are substantially stronger than the effects of inharmonic tonal excitation. Harmonicity is commonly cited as a core acoustic grouping cue, and our results are consistent with this traditional notion. However, our results also suggest that other consequences of harmonic excitation (sparsity, voicing alternation, etc.) are perhaps even more important than harmonicity-driven grouping and provide an example of the insights that can be gained by exploring sound segregation with natural sound signals.

## Methods

### Stimulus generation procedures

Stimuli were generated from speech parameters obtained using a modified version of the STRAIGHT analysis/synthesis procedure^32^. Given a recorded speech utterance, STRAIGHT analysis outputs an estimate of the time-varying f0 (corresponding to voiced excitation), the time-varying spectral parameters of colored noise (corresponding to unvoiced excitation), and the time-varying spectral envelope (corresponding to the vocal tract filter). Each of these estimated generative components can then be manipulated and recombined to synthesize an altered speech signal.

Our stimulus generation used the original STRAIGHT analysis procedures described in ref. ^31^; here we briefly summarize them. All quantities are estimated using a frame rate of 200 Hz and f0-adaptive frame lengths. The estimation procedures for the f0 and spectral envelope are designed to minimize interference between voiced excitation and the spectral envelope due to the sampling of the spectral envelope in time and frequency. Temporal interference is mitigated by averaging power spectra calculated at two time points separated by half of a pitch period. Spectral interference is then eliminated using an f0-adaptive rectangular smoother, followed by post-processing to preserve harmonic component levels (which otherwise would be altered by the partially duplicated smoothing effects of the time windowing for spectrum analysis and the rectangular smoothing of the envelope). The post-processing of the envelope is based on consistent sampling theory^65^. These procedures are implemented with cepstral liftering.

The power ratio between voiced and unvoiced excitation is calculated for each of a set of frequency bands using the residual of the prediction of the bandpass filtered waveform of the pitch period centered on the frame. The prediction is derived from the preceding and succeeding pitch periods. The residual power in these bands determines the parameters of the noise spectrum model (a sigmoid, described by a transition frequency and slope).

STRAIGHT conventionally represents speech excitation as a series of pulses^66^. We employed an alternative version in which speech excitation was modeled sinusoidally, permitting individual frequencies to be manipulated^32^. Specifically, the excitation was defined as a sum of time-varying sinusoids whose frequency contours were proportional to the f0 contour extracted from the speech recording in question:

1 $$v\left( t \right) = \mathop {\sum }\limits_{n = 1}^{N(t)} {\mathrm{cos}}\left( {2\pi \mathop {\scriptstyle\int }\nolimits_0^t a_n\,f_0\left( \tau \right)\mathrm{d}\tau + \varphi _n} \right)$$

For harmonic excitation, *a*_*n*_ = *n*. For inharmonic excitation, *a*_*n*_ deviated from *n* as described below. In all cases, the starting phases *φ*_*n*_ were always 0. *N*(*t*) was adaptively adjusted to keep the highest component frequency below the Nyquist frequency but never exceeded 30 (this upper limit facilitated the selection of inharmonic frequency jitter patterns, and we found informally that excluding harmonics above the 30th did not produce audible effects).

To generate a harmonic or inharmonic speech signal, the sinusoidal excitation and the noise excitation were each divided into frames (10 ms in length, shaped by a Hann window, with 50% overlap between adjacent frames). A minimum-phase finite impulse response (FIR) filter was derived from the spectral envelope at each frame center. The filter was calculated via complex cepstrum using FFT with a 64 ms buffer. The excitation frames were convolved with the minimum phase FIR filter using fast Fourier transform (FFT) convolution, again with a 64 ms buffer (the effective length of the minimum-phase response is <32 ms). Sinusoidal and noise-excited frames for a given time point were added to create a single speech frame. Adjacent frames were combined using the overlap-add method.

Noise-excited stimuli were generated by omitting the sinusoidal excitation used for harmonic/inharmonic synthesis and high-pass filtering the noise excitation to simulate breath noise in whispered speech. The filter was a second-order high-pass Butterworth filter with a (3 dB) cutoff at 1200 Hz whose zeros were moved toward the origin (in the *z*-plane) by 5%. The resulting filter produced noise that was 3 dB down at 1600 Hz, 10 dB down at 1000 Hz, and 40 dB down at 100 Hz, which to the authors sounded like a good approximation to whispering. Without the zero adjustment, the filter removed too much energy at the very bottom of the spectrum.

The noise excitation was combined with the time-varying spectral envelope using the same procedure employed for harmonic and inharmonic speech. The noise-excited stimuli were thus generated from the same spectrotemporal envelope used for harmonic and inharmonic speech, just with a different excitation signal. In this respect, the stimuli differed somewhat from actual whispering, in which speakers may change their articulation relative to normal speaking^67, 68^, and for which the spectral envelope is known to change relative to speech with normal voicing^37, 38^.

In all manipulations, the time-varying spectral envelope was extracted from a recorded utterance and then used to synthesize speech with a variety of excitation signals (harmonic, inharmonic, or noise). Audio was resynthesized at 16 kHz and 16 bits per sample.

Synthesis software is available from the authors upon request.

### Experiment 1 stimuli

Full sentences from the TIMIT database^69^ were synthesized with the harmonics unperturbed or with the third harmonic mistuned upwards by an amount that varied across conditions (0.5, 2, 6, or 20% of the f0, values that pilot demonstrations suggested would span a range of performance levels). Complex tones were synthesized with the first 12 harmonics of an f0 (randomly sampled on a logarithmic scale between 70 and 250 Hz) and were 500 ms in duration. On half the trials, the third harmonic was mistuned (again upwards by 0.5, 2, 6, or 20%). A 10 ms half-Hanning window was applied to the start and end of the tone. All stimuli were presented at 70 dB sound pressure level (SPL) over Sennheiser HD280 headphones via a MacMini with built-in sound card.

### Experiment 1 procedure

The experiment was divided into 10 blocks of 80 trials, each of which presented speech or tones. Speech and tone blocks alternated, counterbalanced in order across participants. On each trial, participants heard a single stimulus and judged whether it was one sound or two concurrent sounds. Different conditions were intermixed randomly within each block (20 trials per condition per block). Half of the trials in each condition did not contain mistuning and were thus indistinguishable across conditions.

The experiment began with a practice session comprised of a block of 20 speech trials and a block of 20 tone trials. Participants were given the option of repeating the practice session if they desired. Feedback was given during the practice session but not during the main experiment. On the practice trials of the speech block, if listeners incorrectly identified a mistuned trial as one sound, they were played the mistuned harmonic in isolation.

### Experiment 1 participants

Ten participants completed the experiment (3 female, mean age = 34.10 years, SD = 13.38 years). Here and elsewhere, all participants had self-reported normal hearing and were native English speakers.

### Experiment 2 stimuli

Inharmonic speech samples were synthesized with jittered harmonic frequencies. The jitter patterns were intended to vary across conditions in the degree to which the harmonic pattern was perturbed, by constraining the perturbation of individual harmonics to a fixed range. Within this range, jitter patterns were selected to be maximally aperiodic subject to the constraint of avoiding low-frequency beating, which we thought might impair intelligibility for reasons unrelated to grouping (by altering the spectrotemporal envelope that conveys speech content). Concurrent pairs of words were always synthesized with the same jitter pattern, to make the relationship between the words analogous to that for harmonic word pairs.

For each condition, we first created 10,000 random jitter patterns where the jitter given to each of the first 30 harmonics other than the f0 component was drawn uniformly from the range [−1 … 1] × *c*, where *c* = 0.1, 0.2, 0.3, or 0.5 (the f0 component was not altered). The *n*th frequency component had the trajectory *j*_*n*_*n*f0(*t*) where *j*_*n*_ is the jitter drawn for the *n*th component. We synthesized complex tones for each of these jitter patterns (30 harmonics, equal amplitude) and measured the height of the largest peak in the autocorrelation function as a measure of periodicity. For each condition (value of *c*), jitter patterns were ranked by their periodicity. To generate the stimuli for a trial, we randomly selected a jitter pattern from the 10 patterns that had the lowest periodicity subject to the constraint of not producing adjacent harmonics that were ever within 30 Hz of each other. To impose the latter constraint, we measured the f0 contours of the utterances used in a trial and then calculated the minimum harmonic spacing based on the minimum f0 (across both utterances for the word pair trials). This ensured that beat frequencies remained above 30 Hz, making them unlikely to interfere with speech structure, which is primarily conveyed by slower modulations^35^. The utterances used on a trial were then synthesized with the same chosen jitter pattern using STRAIGHT. The same jitter pattern was used throughout the inharmonic stimulus used on a trial. Because each frequency component was generated as the f0 contour multiplied by a scalar (taken from the jitter pattern, as in Eq. (1)), they retained the FM of the corresponding harmonic. Harmonics above the 30th were omitted from the resynthesized speech stimulus for all conditions, including the fully harmonic stimuli. Stimuli were presented at 70 dB SPL.

Words were excerpted from TIMIT sentences that were unique to a particular speaker^69^ and that contained at least four words (because the same sentences were used in Experiment 5, which required a minimum of four words per sentence). Excerpted words were constrained to be at least six characters in length. A set of 1200 such words was used for the experiments. These 1200 words were divided into pairs, some of which were used for single-word trials. Each word or word pair was only presented in one condition. Pairs of concurrent words were constrained to be spoken by different speakers of the same gender but from different dialect regions of the TIMIT corpus. Word pairs were then selected to be of similar length subject to these constraints. The mean difference in duration for the two words in a pair was 6.4 ms, with 96% of pairs having a duration difference of <20 ms. Words were excerpted using TIMIT annotations and then windowed with 10 ms onset and offset ramps (half of a Hann window). Concurrently presented words were aligned at their center.

Stimuli for the control experiments (Supplementary Fig. 1) were generated using the same procedure, except that the 30 Hz constraint on the spacing between adjacent harmonics was removed.

### Experiment 2 procedure

For the naturalness rating experiment, participants heard a full sentence and were asked to rate its naturalness on a scale of 1–7 (7 being most natural). Prior to the experiment, they completed a set of 10 practice trials (2 trials per jitter condition) to familiarize them with the range of stimuli in the experiment. Participants were instructed to use the entire scale.

For the intelligibility experiment, participants heard single words or a pair of concurrently presented words. Participants were instructed to type as many words as they could recognize. The experiment took approximately 2 h to complete and consisted of 800 trials. Trials were presented in 4 blocks of 200 trials containing alternating sections of single words and concurrent words: 50 trials of one type, 100 of the other, and then 50 more of the first type. Each section contained equal numbers of harmonic and inharmonic trials, randomly ordered. Blocks alternated between starting with single- or concurrent-word sections, with the starting block type counterbalanced across participants. Each participant completed a total of 800 trials with equal numbers of single- and paired-word trials. Words were randomly assigned to single or paired trials.

To score responses, the experimenter viewed the participant’s response and the TIMIT annotation for each trial and then judged whether the participant had entered the correct word or words (allowing for spelling errors). The experimenter was blind as to the condition when scoring responses, and the same experimenter scored responses across all experiments.

### Experiment 2 participants

Ten participants completed the naturalness experiment (6 female, mean age = 33.1 years, SD = 14.46). Twenty-nine different participants completed the segregation experiment (13 female, mean age = 33.48 years, SD = 10.09).

### Experiment 3 stimuli

Harmonic and inharmonic speech stimuli were generated as in Experiment 2, using *c* = 0.3 (which produced the maximum reduction of intelligibility). Words were presented in silence, speech-shaped noise (SSN), and babble. SSN was generated for each speaker by imposing the average spectrum of each speaker (averaged across all utterances for that speaker in TIMIT) on white noise. Babble was created by summing the resynthesized utterances of 20 randomly selected speakers within a dialect region. We created 40 harmonic and 40 inharmonic exemplars for each dialect region. We randomly drew from these exemplars on each trial of the experiment such that the dialect region of the babble matched the dialect region of the speaker. The genders of the speakers in the babble were random. Babble patterns were created both with the harmonic and jittered stimuli and were crossed with the harmonicity of the word that listeners were to report. We employed a crossed design because it seemed possible that babble generated from inharmonic speech might differ from normal babble in some way that could affect the ease of segregating a foreground utterance (for instance, by having fewer audible glimpses of individual voices). However, there was no obviously audible difference between harmonic and inharmonic babble, and no difference in performance as a function of the babble type, so we combined the data for the analyses we present here. SNRs were selected to produce similar sub-ceiling levels of performance between conditions (−4 dB for SSN and +4 dB for babble); these levels were chosen based on data from a pilot experiment.

### Experiment 3 procedure

Participants were instructed to ignore the background noise when present and type the word that they heard. The experiment took approximately 90 min to complete and contained 576 trials. It was divided into 12 blocks of 48 trials, each subdivided into 3 16-trial subsections, one for each background type (silence, SSN, and babble). Within each subsection, harmonic and inharmonic trials were randomly ordered. There were two blocks with each possible ordering of subsections; block ordering was counterbalanced across participants. Words were drawn from the same set used for Experiment 2, randomly assigned to conditions. The experiment began with 24 practice trials, evenly divided between conditions.

### Experiment 3 participants

Sixteen participants completed the experiment (11 female, mean age = 23.13 years, SD = 7.53).

### Experiment 4 stimuli

The harmonic and inharmonic stimuli were generated as in Experiment 2. Noise-excited stimuli were generated as described in the synthesis methods section above.

Experiment 4A randomly assigned words to conditions as in Experiments 2 and 3, drawn from the same set of word pairs. However, the results revealed that the intelligibility of single words was lower for whispered than for harmonic and inharmonic words. This difference in intelligibility in quiet made it difficult to interpret any differences in intelligibility for speech mixtures. To equate intelligibility in quiet, we ran an additional experiment to determine which words were easiest to understand when whispered and then assigned these to the whispered condition in Experiment 4B.

In this additional experiment used to establish the difficulty of individual words, we presented words in quiet and asked participants to type the word they heard. Half of the words were harmonic and half were noise-excited, with the assignment of words to conditions counterbalanced across participants. Fourteen listeners participated. The outcome of the experiment was an average intelligibility score for each word in each condition. We then created 1,000,000 different random assignments of these words into 4 groups and estimated the average number of words that would be correct in each group for each of the three conditions we sought to include in the main experiment (noise-excited, harmonic, and inharmonic). We used the harmonic intelligibility score for both the harmonic and inharmonic condition on the assumption (based on data from Experiments 2 and 3) that performance would be approximately equal for those two conditions. We chose the word-condition assignment for which average intelligibility was most similar across conditions. In practice, the words chosen for the whispered condition were those that were easiest to understand. This procedure evaluated random splits into four subsets (only three of which were actually used, because there were three conditions in the main experiment) because we found that splitting into three subsets did not produce small enough word sets to equate performance across conditions. The resulting subsets of words were then assigned to word pairs using the same procedure described for Experiment 2, a third of which were used for the single-word trials (assignment counterbalanced across participants).

### Experiment 4 procedure

Experiment 4A took 1.5 h and was 600 trials long. It was blocked into sets of single- or double-word trials, but the blocks were shorter than in other experiments (60 trials total, either 10 single words, 40 word pairs, and 10 single words or 20 word pairs, 20 single words, and 20 word pairs, with the two block types alternating, and order counterbalanced across participants). The experiment contained two conditions not analyzed here.

Experiment 4B took 1.5 h and was 600 trials long. In all other respects, the procedure was identical to that of Experiment 2 in blocking by single word or word pairs (blocks of 30, 60, and 30 trials, either single–concurrent–single or concurrent–single–concurrent); harmonic/inharmonic/whispered trials were randomly ordered within the blocks.

### Experiment 4 participants

Twenty participants completed Experiment 4A (16 female, mean age = 20.9 years, SD = 2.59). Eight participants completed Experiment 4B (4 female, mean age = 31.88 years, SD = 14.82).

### Experiment 5 stimuli

Harmonic, inharmonic, and whispered utterances were generated as in Experiments 2–4, using a jitter amplitude of 0.3. The whispered stimuli were not equated for difficulty, because for sentences, there was not much variation in intelligibility in quiet across conditions. Sentences were selected from the set from which words were excerpted in Experiments 2–4 (containing at least four words, at least one of which was at least six characters long). Sentences from this set were paired by selecting sentences by speakers of the same gender from different dialect regions whose lengths were as similar as possible (mean duration difference = 15.6 ms, with 95% within 40 ms of each other). Concurrent sentences were aligned at their centers. Only those TIMIT sentences that were unique to a speaker were used, to avoid priming effects that might result from hearing the same sentence more than once.

### Experiment 5 procedure

At the start of a trial, the first two words of the cued sentence appeared on the computer screen; participants were instructed to listen to the sentence that began with those two words and to type in the last two words of that sentence. Responses were scored separately for the cued and uncued sentence (the experimenter viewed the response for a trial along with the last two words of the cued sentence, and then the last two words of the uncued sentence, and evaluated the number of correctly entered words in each case, allowing for spelling errors). The scoring was performed blind as to the condition.

The experiment took 2 h to complete and was 600 trials long. Trials were separated into blocks of 60 trials containing sections of trials with single sentences or concurrent sentences. Blocks were structured in one of the two ways: 10 single sentence trials, 40 concurrent sentence trials, and 10 single sentence trials, or 20 concurrent sentence trials, 20 single sentence trials, and 20 concurrent sentence trials. These two types of blocks alternated and ordering was counterbalanced across participants. Each subsection within a block contained equal numbers of harmonic, inharmonic, and whispered trials. Single sentences formed only 1/3 of trials because participants performed close to ceiling on them.

### Experiment 5 participants

Fifteen participants completed the experiment (8 female, mean age = 28.20 years, SD = 9.61).

### Experiment S1 stimuli

Stimuli were generated exactly as in Experiment 2 but without the 30 Hz beating constraint on frequency jittering.

### Experiment S1 procedure

The procedure was identical to that of Experiment 2.

### Experiment S1 participants

Fifteen participants completed the naturalness experiment (6 female, mean age = 39.67 years, SD = 15.69). Fifteen participants completed the word recognition experiment (6 female, mean age = 37.0 years, SD = 10.72), 6 of whom also completed the naturalness experiment.

### Ethics

All experiments were approved by the Committee on the Use of Humans as Experimental Subjects at the Massachusetts Institute of Technology. Informed consent was obtained from all participants.

### Sample sizes

Pilot versions of all experiments except Experiment 4A were run beforehand (Experiment 4A was the pilot experiment for Experiment 4B). Sample sizes were chosen based on the effect sizes in these pilot experiments. These pilot experiments also provided replications (with minor differences) of each experiment. Each effect described in the paper was replicated at least once.

### Statistics

*t*-Tests and repeated-measures analyses of variance were used to test for differences in performance between conditions and for interactions between conditions. Mauchly’s test was used to test for violations of the sphericity assumption. Data distributions were assumed to be normal and were evaluated as such by eye.

### Code availability

The code used to generate the stimuli is available from the corresponding author upon reasonable request.

### Data availability

The data that support the findings of this study are available as a supplementary file.

## Electronic supplementary material

Supplementary Information

Description of Additional Supplementary Information

Dataset 1
