# Supplementary material for: Inharmonic speech reveals the role of harmonicity in the cocktail party problem
Source: Nat Commun. 2018 May 29;9:2122. doi: 10.1038/s41467-018-04551-8 (PMC5974276; doi:10.1038/s41467-018-04551-8)
Supplement: Supplementary file 1 — Supplementary Information [file 41467_2018_4551_MOESM1_ESM.pdf]

# Supplementary Information

## Inharmonic speech reveals the role of harmonicity in the cocktail party problem

Popham et al.

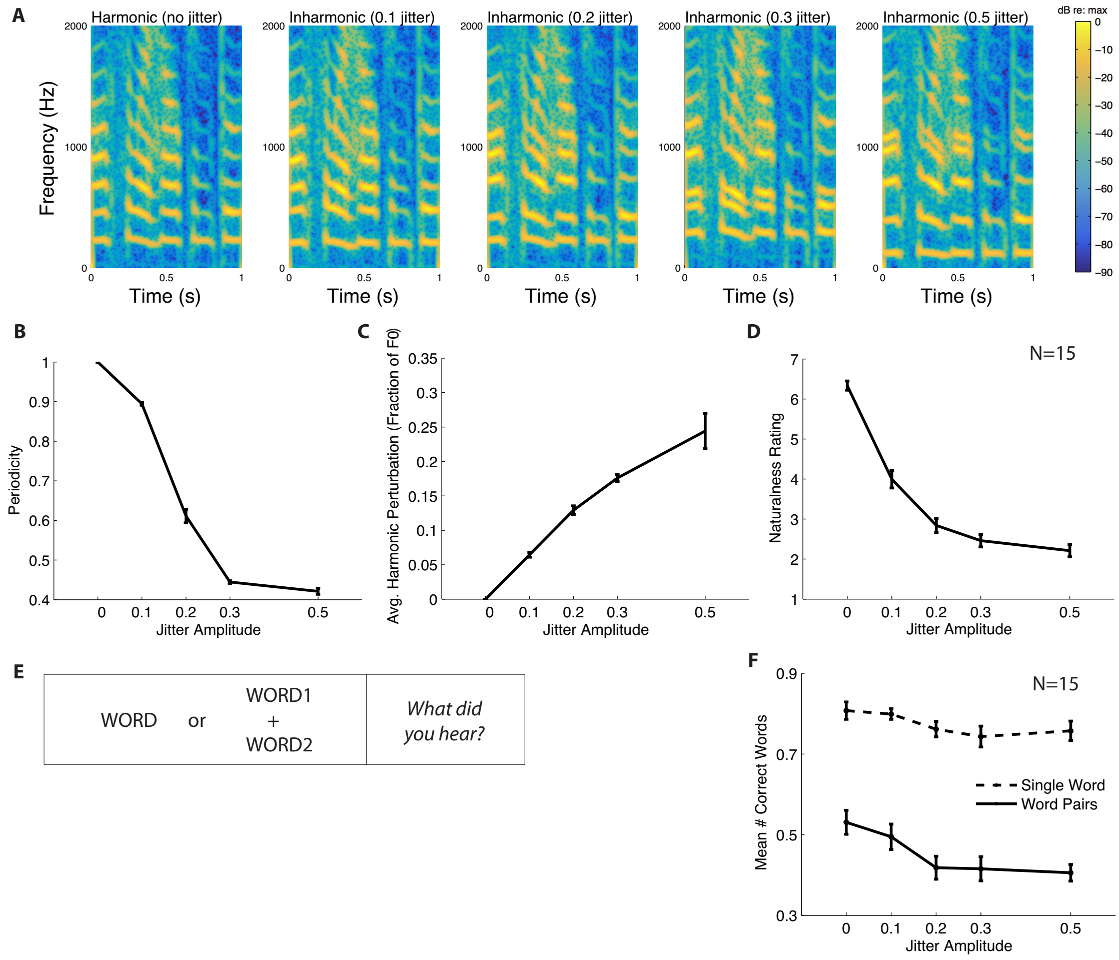


**Supplementary Figure 1**. Results of parametric inharmonicity manipulation with jitter patterns unconstrained by the minimum frequency spacing (Experiment S1). A. Spectrogram of excerpt of harmonic and inharmonic utterances with harmonics jittered by 10%, 20%, 30%, and 50% of the F0, respectively. B. Waveform aperiodicity (the height of the peak of the autocorrelation function) for each jitter condition. C. Average absolute harmonic perturbation for each jitter condition. Error bars here and in (B) plot standard deviation. D. Ratings of naturalness of spoken sentences for different degrees of inharmonicity (N=15). There was a modest difference in naturalness between the .3 and .5 jitter conditions, unlike when the jitter was constrained to minimize beating ($t(14) = 3.36$, $p=0.0047$). E. Schematic of trial structure for word segregation task (same as in Experiment 2). F. Intelligibility of single words and concurrent word pairs as a function of the degree of inharmonicity (N=15). Unlike when jitter was constrained to minimize beating, the jitter manipulation affected intelligibility of single words in quiet ($F(4, 56) = 4.7415$, $p=0.0023$). However, the effect of inharmonicity on mixture intelligibility again leveled off once the jitter magnitude exceeded .3 ($t(14) = 0.5$, $p=0.63$), suggesting that the limited effect of inharmonicity in Experiment 2 was not an artifact of the constraint imposed on the frequency jitter. Error bars here and in (D) plot SEM.
