# Supplementary material for: Inharmonic speech reveals the role of harmonicity in the cocktail party problem
Source: Nat Commun. 2018 May 29;9:2122. doi: 10.1038/s41467-018-04551-8 (PMC5974276; doi:10.1038/s41467-018-04551-8)
Supplement: Supplementary file 2 — Description of Additional Supplementary Information [file 41467_2018_4551_MOESM2_ESM.pdf]

Description of Additional Supplementary Files

File Name: Supplementary Data 1
Description: This file contains all the data used to generate the results figures in the paper.
